# Establishment and Long-Term Expansion of Small Cell Lung Cancer Patient-Derived Tumor Organoids

**DOI:** 10.3390/ijms22031349

**Published:** 2021-01-29

**Authors:** Seon Young Choi, Yong-Hee Cho, Da-Som Kim, Wonjun Ji, Chang-Min Choi, Jae Cheol Lee, Jin Kyung Rho, Gi Seok Jeong

**Affiliations:** 1 Asan Institute for Life Sciences, Asan Medical Center, Seoul 05505, Korea; vet904rainbow@gmail.com (S.Y.C.); bioyonghee1@naver.com (Y.-H.C.); 2 Department of Biomedical Sciences, Asan Medical Center, AMIST, College of Medicine, University of Ulsan, Seoul 05505, Korea; som5060@hanmail.net; 3 Department of Pulmonology and Critical Care Medicine, Asan Medical Center, College of Medicine, University of Ulsan, Seoul 05505, Korea; jack1097@naver.com (W.J.); ccm9607@gmail.com (C.-M.C.); 4 Department of Oncology, Asan Medical Center, College of Medicine, University of Ulsan, Seoul 05505, Korea; jclee@amc.seoul.kr; 5 Department of Convergence Medicine, Asan Medical Center, College of Medicine, University of Ulsan, Seoul 05505, Korea; 6 Biomedical Engineering Research Center, Asan Institute for Life Sciences, Asan Medical Center, Seoul 05505, Korea

**Keywords:** small cell lung cancer, patient-derived tumor organoids, patient avatar model systems

## Abstract

Differential chemo-sensitivity of cancer cells, which is attributed to the cellular heterogeneity and phenotypic variation of cancer cells, is considered to be the main reason for tumor recurrence after chemotherapy. Here, we generated small cell lung cancer patient-derived tumor organoids and subjected them to long-term expansion with the addition of WNT3A or R-spondin1. We confirmed that the organoids have similar genetic profiles, molecular characteristics, and morphological architectures to the corresponding patient tumor tissue during and after long-term expansion. Interestingly, the cellular heterogeneity of organoids is reflected in their differential response to cisplatin or etoposide. We propose to utilize the organoids as small cell lung cancer patient avatar models that would be ideal for investigating the mechanisms underlying tumor recurrence after chemotherapy, and would ultimately help to develop personalized medicine.

## 1. Introduction

Lung cancer is the leading cause of cancer-related mortality worldwide. It can be broadly classified into non-small cell lung cancer (NSCLC) and small cell lung cancer (SCLC), comprising approximately 80–85% and 15–20% of all lung cancers, respectively [1,2]. The two classes display histopathological and molecularly distinct characteristics [3]. Compared with NSCLC, SCLC shows a rapid doubling time, is highly malignant, and typically metastasizes early and more widely [2]. Although the overall survival rate of patients with lung cancer has improved due to newly developed anti-cancer agents against NSCLC, the same is not true for SCLC, and it is therefore still considered one of the most lethal cancer types worldwide. Indeed, the poor prognosis of SCLC is exemplified by the absence of any significant advances in first line therapy since the introduction of the current standard etoposide–platinum doublet (EP). Although most patients with SCLC (60–70%) are responsive to initial EP therapy, the majority display a poor prognosis after EP chemotherapy due to inevitable tumor recurrence [4]. The development of anti-cancer agents for treating SCLC is therefore essential.

Recent sequencing studies on several sub-regions of primary tumors and metastatic cancer tissues revealed an unexpected degree of inter-tumoral and intra-tumoral heterogeneity. The degree of intra-tumoral heterogeneity was found to be highly variable as measured by coding mutations [5,6]. Moreover, single-cell RNA sequencing analyses after anti-cancer agent treatment indicated that the clonal diversity was associated with parallel evolution tracts that enabled the selection and expansion of surviving clones [7,8]. Given the close relationship between cancer cell heterogeneity and drug sensitivity, investigation into patients with SCLC after EP therapy using model systems that better represent the patients’ unique heterogeneous cancer cell populations is needed. This would be an improvement on conventional cancer cell lines, which have fundamental limitations, including a failure to represent the cellular heterogeneity of the corresponding tumor tissue [9,10,11,12].

At present, the most common models used to represent the heterogeneity of human cancer cells are patient-derived tumor organoids (PDTOs) [13,14,15,16] and patient-derived tumor xenografts (PDTXs). Both model systems recapitulate the original tumor structure and have similar molecular characteristics to their matched tissues. Of the two, the PDTX model system exhibits a tumor microenvironment more similar to the original tumor tissue and also allows researchers to validate their studies in vivo. However, it has a low success rate, is labor-intensive, and requires many months of work, making it unsuitable for individualized therapy on a large scale [17,18]. Recently, intense efforts have focused on generating tissue-specific PDTOs, including those for colon, pancreas, prostate, liver, breast, and bladder cancer, and novel extracellular membrane components as scaffolders have been developed [16,19,20,21,22,23,24]. Lung cancer PDTOs, mostly consisting of NSCLC [13,25], have been established that successfully recapitulate the original cancer tissue architecture. However, the culture conditions specific for the long-term survival of SCLC PDTOs have not yet been fully optimized.

In the current study, we generated tumor organoids using cells from patients with SCLC that recapitulate the architecture of the original tumor tissues. Further, we investigated whether combinations of Wnt3A or R-spondin1 in epidermal-growth factor (EGF) and fibroblast growth factor (FGF)-containing culture conditions promoted the long-term survival of SCLC organoids. The results indicated the pivotal role of the Wnt/β-catenin pathway in the long-term expansion of SCLC when Wnt3A or R-spondin1 was added to the culture media. The morphology, molecular characteristics, and genomic profiles of SCLC organoids were similar to those of the original tumor tissues from matched patients. The organoids consisted of cells that differed in their responsiveness to cisplatin and etoposide treatment. The response of SCLC patients to cisplatin and etoposide was represented by their matched PDTOs. Thus, we suggest that SCLC PDTOs expanded in the presence of Wnt3A or R-spondin1 could survive for a long time and could be a powerful tool to develop an effective anti-cancer therapy for SCLC.

## 2. Results

### 2.1. Establishment of PDTO Culture Systems Optimized for SCLC

**Figure 1:**
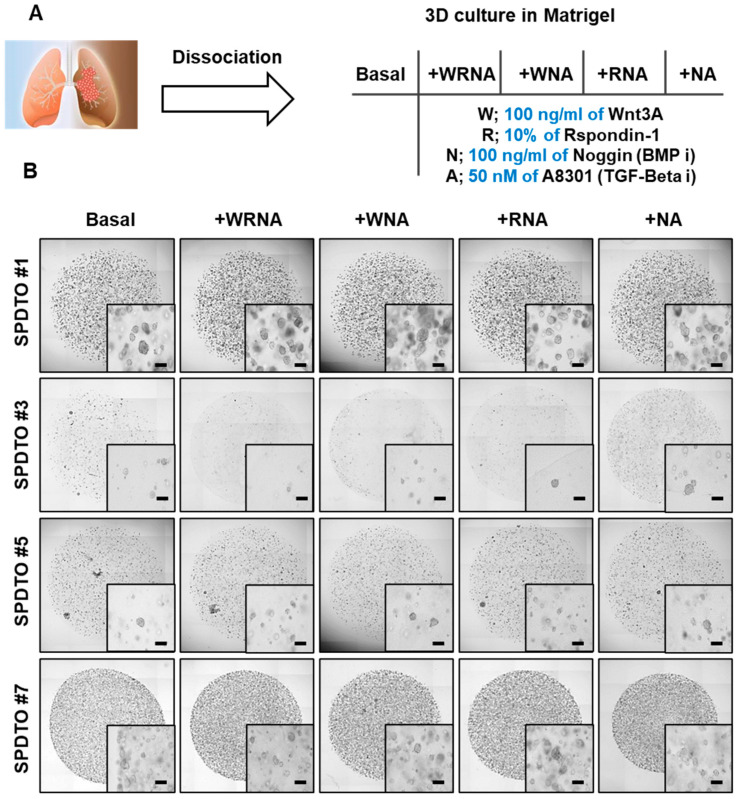
Establishment of tumor organoids derived from small cell lung cancer. (**A**) Tumor tissues were obtained from patients with small cell lung cancer (SCLC) and cultured under various conditions to form organoids. (**B**) Brightfield images of SPDTO formation in short-term culture under five different culture conditions from passage 3 to passage 6. Basal: media containing EGF (50 ng/mL) and FGF (50 ng/mL). WRNA: media containing Wnt3A (100 ng/mL), R-spondin1 (10%), Noggin (100 ng/mL), and A8301 (50 nM). WNA: media containing Wnt3A, Noggin, and A8301. RNA: R-spondin-1, Noggin, and A8301. NA: media containing Noggin and A8301. Images of organoids cultured in various media after passaging (passage 6) (Scale bars, 200 µm).

Despite the fact that lung cancer PDTOs have been established in recent studies, they mostly represent non-small cell lung cancer (NSCLC) [13]. Given the distinct characteristics of SCLC and NSCLC, we sought to optimize the culture conditions for SCLC PDTOs (SPDTOs). Fresh biopsied samples were obtained from tumor tissues from patients with SCLC (Table 1), and were cultured using previously adapted culture conditions (basal; EGF, bFGF in Matrigel) [13] and using four additional culture conditions, comprising different combinations of 100 ng/mL Wnt3A, 10% R-spondin1 conditioned medium, 100 ng/mL Noggin, and 50 nM A8301 (Figure 1A and Table 1). We successfully generated SCLC organoids from 8 of the 10 SCLC tumor samples, using five different culture conditions (Table 1 and Figure 1B). Initial SCLC organoid formation varied between patient samples, with some tumors forming organoids with a high frequency, while others yielded a very low frequency of primary organoids. This difference is likely due to the presence of heterogeneous cell populations within the tumors, and the fact that the biopsied regions may contain cells with different proliferation rates, and maybe even some necrotic cells. The growth rates of some organoids, such as SPDTO #3, decreased over time; these were therefore not suitable for investigating SCLC. In addition, the different culture compositions did not affect the initial formation of SPDTOs. However, the addition of Noggin, an inhibitor of epithelial cell differentiation, and A8301, an inhibitor of growth arrest after limited population doubling, to the basal culture mildly augmented the plating efficiency of SCLC organoids (Figure 1B). In contrast, other factors, such as R-spondin1 and Wnt3A, did not show differences from or synergistic effects with Noggin- or A8301-containing basal media, with respect to the plating efficiency of SCLC organoids (Figure 1B). The calculated numbers and sizes of SPDTOs (as the sum of each value for the number and size of SPDTOs from passage 3 to passage 6) in short-term culture were not significantly different (data not shown). These results suggest that the ALK receptor and BMP signaling may negatively regulate the initial formation of SPDTOs.

### 2.2. Optimization of Culture Conditions for Long-Term Viability of SCLC Cells 

**Figure 2:**
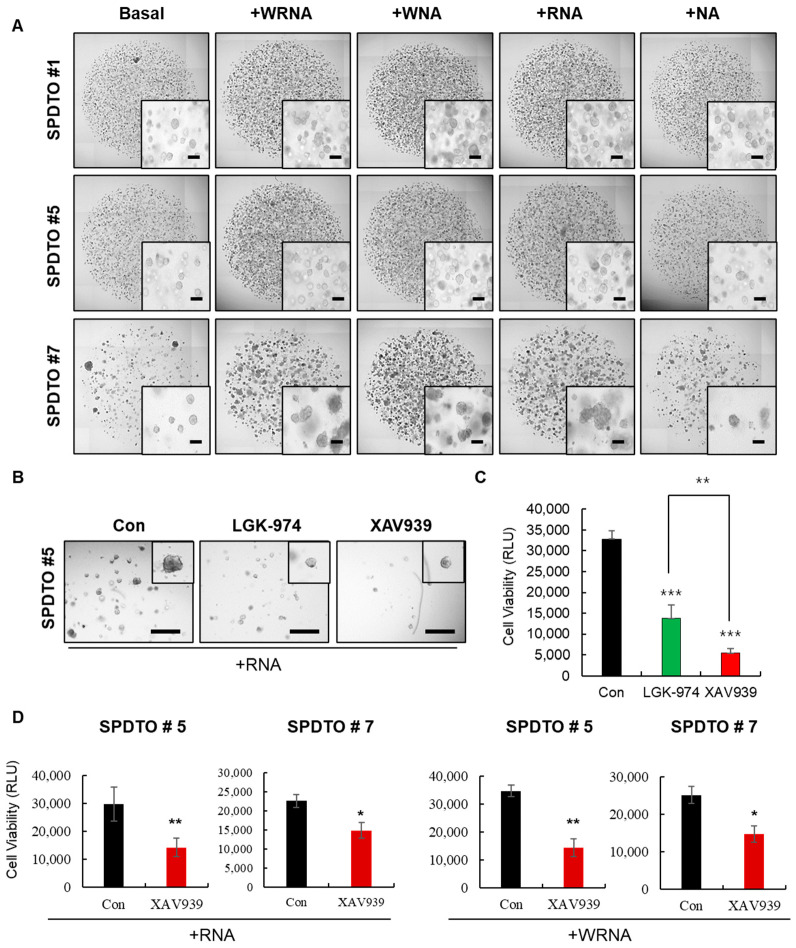
Long term-expansion of SCLC organoids after addition of Wnt3A or R-spondin1. SPDTOs were grown under 5 different culture conditions over 9 months. (**A**) Brightfield images of SPDTO formation in long-term culture under five different culture conditions from passage 7 to passage 12. Basal: medium containing EGF (50 ng/mL) and FGF (50 ng/mL). WRNA: medium containing Wnt3A (100 ng/mL), R-spondin1 (10%), Noggin (100 ng/mL), and A8301 (50 nM). WNA: medium containing Wnt3A, Noggin, and A8301. RNA: R-spondin-1, Noggin, and A8301. NA: medium containing Noggin and A8301. The images are of organoids cultured in the various media after passaging (passage 12) (Scale bars, 200 µm). (**B**,**C**) Brightfield images of SPDTO #5 grown with RNA in the presence of 5 µM LGK-974 or 10 µM XAV939 (B) and quantification via cell titer assay. Scale bars represent 650 µm (C). (**D**) SPDTO #5 or SPDTO #7 were grown with 10 µM XAV939 or DMSO under RNA or WRNA culture conditions. Significance denoted as n.s. not significant, * *p* < 0.05, ** *p* < 0.01, or *** *p* < 0.001. Data were obtained from at least three independent experiments. Con, control.

Given the fact that decreased viability and tissue degradation are likely to occur during long-term culturing under sub-optimal conditions, we sought to determine the culture conditions most suitable for the long-term culture of SCLC cells. We cultured SPDTOs for more than nine months using five different culture conditions. We found that Wnt3A, a major canonical Wnt/β-catenin pathway-activating ligand, and R-spondin1, a stem cell-specific Wnt/β-catenin agonist, had critical roles in the long-term maintenance of SCLC tumor organoids (Figure 2A). However, the synergistic effects of R-spondin1 and Wnt3A were not observed, as shown by the normal organoid-forming ability with the WRNA culture condition (Figure 2A). The basal culture conditions used for short-term organoid formation (Figure 1B) do not maintain the long-term viability of SCLC tumor organoids, and this was not improved by the addition of Noggin or A8301 (Figure 2A). The calculated numbers of organoids in SPDTO #5 (the sum of each value from passage 7 to passage 12) in long-term cultures were significantly different in the +WRNA conditions, compared to those in the presence of basal medium; the numbers of organoids in SPDTO #7 were significantly different in the +WRNA, +WNA, and +RNA conditions, compared to those in the presence of basal medium (data not shown). However, no repeat experiments were performed for each passage. Treatment with LGK-974 and XAV939, which inhibit the Wnt ligand and Wnt/β-catenin pathways, respectively, suppressed the organoid-forming efficiency of SPDTO #5 cells grown under RNA culture conditions (Figure 2B,C). Similarly, XAV939 treatment effectively inhibited organoid formation in SPDTO #5 and SPDTO #7, which were grown under RNA and WRNA culture conditions, respectively (Figure 2D). These results indicate that the basal culture conditions previously determined for lung cancer PDTOs are not suitable for the long-term maintenance of SPDTOs, and that the inhibition of ALK receptor and BMP signaling is not sufficient to maintain long-term SCLC tumor organoids. Rather, the activation of the Wnt/β-catenin pathway by the addition of WNT3A or R-spondin1 is a prerequisite for the long-term expansion of SCLC tumor organoids, in combination with ALK receptor and BMP signaling inhibition. 

### 2.3. Characterization of SCLC Tumor Organoids as a Patient Avatar Model

**Figure 3:**
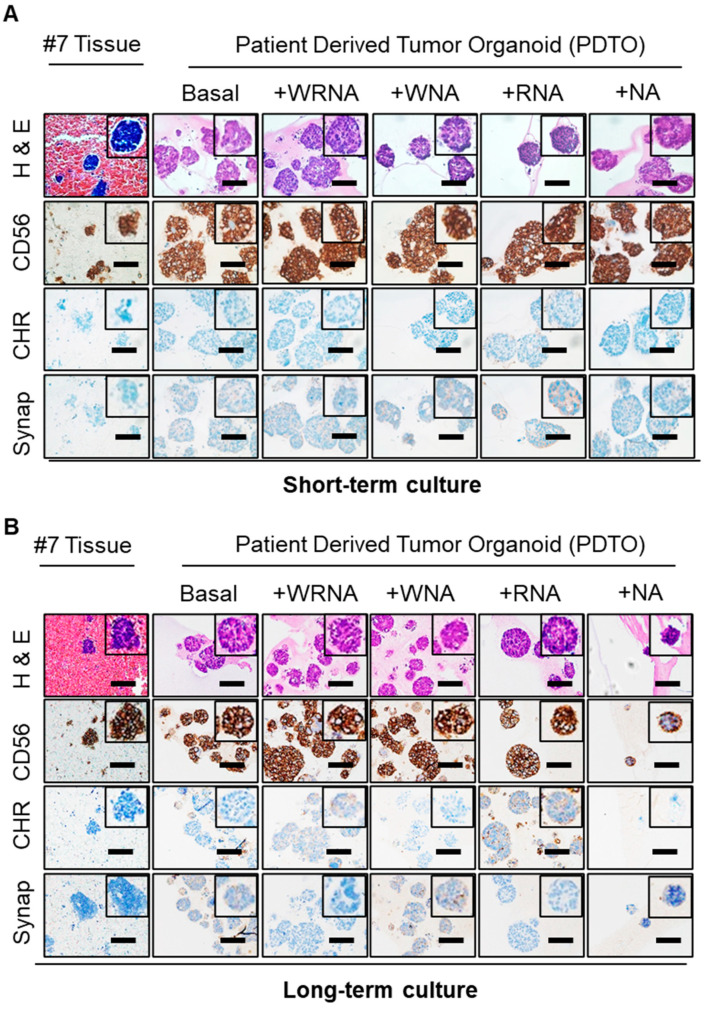
Essential role of the Wnt/β-catenin pathway in long-term expansion. Hematoxylin and eosin (H&E) and immunostaining of organoids grown under culture conditions as indicated, with corresponding tumor sections. (**A**) H&E and immunostaining analysis of SPDTO #7 performed using organoids grown under 5 different culture conditions after short-term culture, together with matched tumor tissue. (**B**) H&E and immunostaining of SPDTO #7 performed using organoids grown under 5 different culture conditions for 9 months, together with matched tumor tissue. Scale bars represent 100 µm.

To identify the appropriate culture conditions for SCLC organoids such that they represent the original tumor tissue, we compared the genetic profiles and morphological and molecular characteristics of organoids cultured under five different conditions and their corresponding tissues. Hematoxylin and eosin (H&E) staining revealed that regardless of the culture conditions, SPDTO #5 and #7 showed a similar morphology to their corresponding tumor tissue in both long-term and short-term culture (Figure 3a and Supplementary Figure S1a). Similarly, the molecular characteristics of the SCLC tumor tissues were consistent with their matched SPDTOs under all culture conditions, as shown by immunohistochemical (IHC) analyses of CD56, chromogranin, and synaptophysin (Figure 3a and Supplementary Figure S1a). In addition, these similarities were not altered during the long-time expansion of SCLC organoids (Figure 3b and Supplementary Figure S1b), suggesting that the culture conditions do not affect the molecular and morphological characteristics of SPDTOs. These data indicate that ALK receptor, Wnt/β-catenin, and BMP signaling do not play a critical role in recapitulating the corresponding SCLC tissues and do not affect genomic stability. 

To evaluate genomic similarities, genomic DNA was isolated from primary patient tumor tissues and the corresponding organoids grown for nine months after initial organoid formation under five different culture conditions and subjected to whole exome sequencing (WES). Results showed that SPDTO #5 and #7 grown for nine months under any of the five different culture conditions retained a genetic profile similar to that of the corresponding patient SCLC tissue (Supplementary Figure S2), suggesting that the culture conditions do not affect mutational status or genomic stability after long-term expansion.

### 2.4. SCLC Organoid Proof-of-Concept Drug Screen

**Figure 4:**
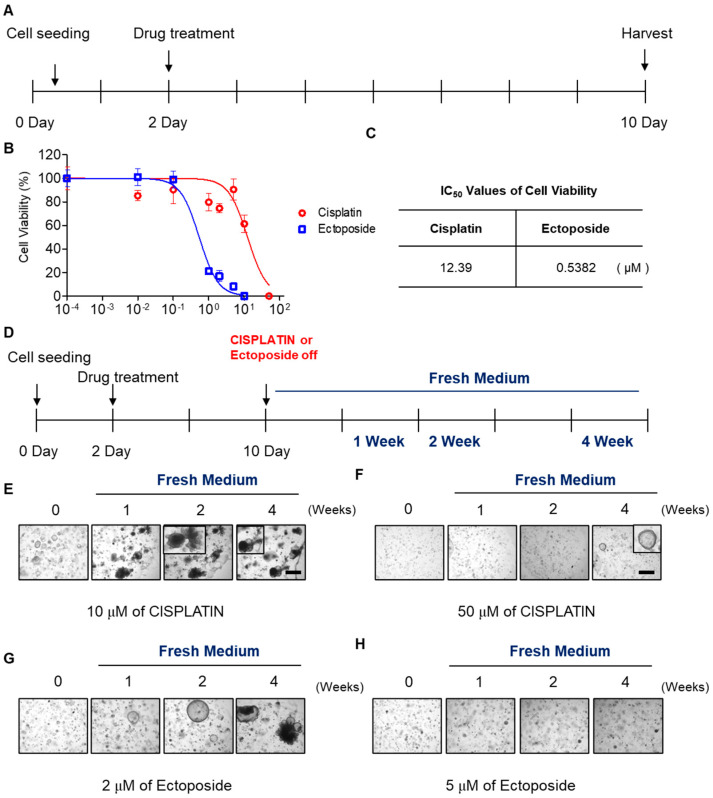
Development of SPDTO as a drug screening assay. SPDTO #7 was treated with various concentrations of cisplatin or etoposide for 8 days then organoids were transferred to fresh medium for 4 weeks. (**A**) Cisplatin or etoposide treatment schedules. (**B**) IC_50_ graph of cisplatin and etoposide with SPDTO #7. (**C**) IC_50_ values for cisplatin and etoposide with SPDTO #7 (**D**). Schedules for monitoring SPDTO #7 after withdrawal of cisplatin or etoposide for 4 weeks, monitored in fresh medium at 1, 2, and 4 weeks. (**E**–**H**) Bright-field images of SPDTO #7 grown in fresh medium after treatment with 10 µM cisplatin (**E**), 50 µM cisplatin (**F**), 2 µM etoposide (**G**), or 5 µM etoposide (**H**). Scale bars represent 75 µm.

It is important to identify novel anti-cancer agents to treat SCLC. To this end, we sought to develop a model system using SCLC organoids that could serve as a drug screening tool. We used cisplatin or etoposide to treat SPDTO #7 and SPDTO #10 grown under RNA culture conditions to test whether SCLC organoids would be suitable to use as a drug screening system (Figure 4A; Supplementary Figure S3A). The therapeutic effects of cisplatin and etoposide on SPDTO #7 and SPDTO #10 were evaluated by measuring the half maximal inhibitory concentration (IC50) and the slope of the dose–response curve. The viability of SCLC organoids was reduced by treatment with cisplatin or etoposide in a dose-dependent manner (Figure 4B). SPDTO #7 was more sensitive to etoposide (0.5382 µM) than cisplatin (12.39 µM) as measured by IC50 values (Figure 4C). This differential sensitivity was unexpected; therefore, we sought to explore whether these SDPTOs would be suitable for investigating cancer recurrence. We withdrew the cisplatin or etoposide treatment after 10 days, and continued to change the media of the organoids for a further 4 weeks (Figure 4D). At a dose of 10 µM cisplatin, some cells did not respond to the cisplatin and the growth of the organoids was not suppressed (Figure 4E). Moreover, after the removal of cisplatin, the organoids expanded and were fully grown 2 weeks after drug withdrawal (Figure 4E). Several SCLC cells within the organoids survived treatment with 50 μM cisplatin, and grew abruptly 4 weeks after cisplatin removal. Similarly, the SCLC cells that survived treatment with 2 µM etoposide for 10 days also started to form organoids within 1 week, and showed maximal growth 2 weeks after drug withdrawal (Figure 4F). However, organoid formation did not occur when doses of etoposide above 5 µM were used (Figure 4G). These results demonstrate that our optimized culture conditions for generating SCLC organoids could be used as a drug screening system because they retain the differential sensitivity of SCLC cells to EP therapy.

## 3. Discussion

Recent evidence has suggested that the cancer cell lines commonly used in cancer research do not represent a suitable model for the development of novel anti-cancer agents [9,10,11,12]. For instance, a direct relationship between sensitivity to a specific drug and a single genomic alteration has not been demonstrated. Instead, complex interacting factors between multiple genomic alterations are difficult to identify, and cancer cells typically comprise a heterogeneous population. Therefore, it remains a challenge to develop algorithms of genomic alterations in the context of a unique genetic background. Given that tumors are composed of a variety of sub-clones associated with clonal evolution via Darwinian selection, cellular heterogeneity and phenotypic variation allows the emergence of a complex clonal architecture, which is reflected in pathological features such as drug resistance and recurrence potential. The development of suitable models representing factors that affect tumor heterogeneity and evolution, and how heterogeneity impacts drug response, will be important to fully exploit their potential for predicting patient responses to treatment.

The present study describes protocols for deriving SCLC organoids that allow the long-term culture of patient-derived tumor cells. In contrast to the short-term culture of SCLC tumor cells, R-spondin1 and Wnt3A are essential factors for the long-term culture of SCLC tumor organoids. Wnt3A is known to a play critical role in the maintenance of epithelial stem cells via the activation of Wnt/β-catenin signaling, and R-spondin1, the ligand for LGR5, specifically enhances the Wnt/β-catenin pathway. The activation of the Wnt/β-catenin pathway in SCLC organoids is essential for their maintenance during long-term expansion. We also observed that SCLC cells grown with XAV939, a potent Wnt pathway inhibitor, significantly limits the success of their long-term culture. However, the addition of LGK-974, an inhibitor of the Wnt ligand, has a marginal effect on SCLC cells during the long-term expansion of SPDTOs. These results clearly indicate that the addition of extrinsic Wnt-activating components, such as R-spondin1 or WNT3A, is essential for the long-term expansion of SPDTOs.

In order to continue to advance the field of cancer medicine, detailed clinical investigations are essential.

Although the use of organoids to completely mimic human cancer biology lacking microfluidics has several limitations [26], organoid systems reflect the effects of interactions between heterogeneous cancer cells. In addition, they include categorizing patients according to molecular characteristics of the tumor cells and seeking the optimal anti-cancer agent or drug combination for each. Given the fact that SCLC organoids retain the genetic features and molecular/histological characteristics of the corresponding patient-derived tumor tissues, these organoids could be valuable for advancing cancer medicine. In addition, organoid systems overcome the current homogenous and 2D culture systems. The heterogeneous SCLC organoids that exhibit differential responsiveness to cisplatin or etoposide would be useful for overcoming the current problem of SCLC recurrence in patients after EP therapy. Moreover, in the context of long-term expansion availability and variable drug responsiveness, SCLC organoids could represent a novel platform for drug screening and clinical trials.

## 4. Materials and Methods

### 4.1. Tumor Tissue Preparation and Organoid Establishment

Biopsied tumor samples were obtained from patients with SCLC after receiving patient-informed consent and approval from the IRB of Asan Medical Center (2019-1139). All studies involving human participants were conducted in accordance with the International Ethical Guidelines for Biomedical Research Involving Human Subjects. Clinical diagnosis of SCLC was validated by pathologic review. Organoid cultures were performed as previously reported [13], with some modifications. Tumor tissues were washed with cold Hank’s buffered salt solution (HBSS) containing antibiotics and sectioned using sterile blades. Sectioned samples were incubated with 0.001% DNase (Sigma-Aldrich, St. Louis, MO, USA), 1 mg/mL collagenase/dispase (Roche, IN, USA), 200 U/mL of penicillin, 200 mg/mL of streptomycin in Advanced DMEM/F12 medium (Gibco, OK, USA) at 37 °C for 15 min with intermittent agitation. After incubation, suspensions were repeatedly triturated by pipetting and passed through a 100 μm cell strainer (BD Falcon, CA, USA). Cells were then centrifuged at 112× *g* for 3 min, and the pellet was resuspended in 100 μL basal medium (Advanced DMEM/F12) supplemented with 20 ng/mL of bFGF (Invitrogen, CA, USA), 50 ng/mL of human EGF (Invitrogen, CA, USA), 1× N2 (Invitrogen, CA, USA), 1× B27 (without vitamin A, Invitrogen, CA, USA), 10 μM of Y27632 (Tocris, Bristol, UK), and 1% penicillin/streptomycin (Gibco, OK, USA). Then, 100 μL of Matrigel (Corning, NY, USA) were added to the remaining 50 μL suspension to establish SCLC organoids, and the resulting cell suspension was allowed to solidify on pre-warmed 6-well culture plates (SPL, Korea) at 37 °C for 10 min. After gelation, 3 mL basal medium was added to the well. Medium was changed every 2–3 days, and organoids were passaged for 2–3 weeks. For passaging, a solidified Matrigel drop containing the SCLC organoids was harvested using cold DPBS then centrifuged at 112× *g* for 3 min at 4 °C. Pellets were washed with cold DPBS and centrifuged at 250 rcf for 15 min at 4 °C. Organoids were resuspended in 2 mL TrypLE Express (Invitrogen, CA, USA) and incubated for 10 min at 37 °C for dissociation. Afterwards, 10 mL basal medium was added and centrifuged at 112× *g* for 3 min. Pellets were washed with DPBS and centrifuged at 112× *g* for 3 min then resuspended in basal medium and reseeded at a 1:5 ratio to allow the formation of new organoids in passage 1 and passage 2.

To isolate cells from the organoid for passage, after passage 2, the organoids that had formed in Matrigel were dissociated with TrypLE Express for 10 min at 37 °C to prepare single cells. A drop of Matrigel (40 µL) containing 5000 cells was added to a well of a pre-warmed 24-well culture plate (SPL, Gyeonggi-do, Korea) to test the organoid-forming efficiency of the various culture conditions. The basal condition comprised advanced DMEM/F12, 1× N2, 1× B27 (without vitamin A), 20 ng/mL basic FGF, 50 ng/mL human EGF, 10 µM Y27632, and 1% penicillin/streptomycin. The +WRNA condition comprised the basal medium supplemented with 100 ng/mL Wnt3A (R&D systems), 10% R-spondin 1 conditioned medium, 100 ng/mL Noggin (Peprotech, NJ, USA), and 50 nM A8301 (Tocris, Bristol, UK). The +RNA condition comprised basal medium supplemented with 10% R-spondin 1, 100 ng/mL Noggin, and 50 nM A8301. The +NA condition comprised basal medium supplemented with 100 ng/mL Noggin and 50 nM of A8301. Organoid-forming efficiency was measured by quantification of organoid numbers and size, as described previously [14]. Eight-bit binary images of whole organoid drops were analyzed by Image J software (NIH) using the “analyze particle” option set to the following parameters: size, 25-infinite and circularity 0.3–1.0 (short-term culture: ~passage 6; long-term culture: ~passage 12).

### 4.2. Immunohistochemistry

Four-micrometer-thick paraffin-embedded tissue samples and organoids were incubated with 10 mM sodium citrate buffer (pH 6.0), and autoclaved for 15 min for antigen retrieval. Samples were then blocked with PBS containing 5% bovine serum albumin (BSA; Affymetrix, CA, USA) and 1% normal goat serum (NGS, Vector Laboratories, CA, USA) for 30 min. After blocking, sections were incubated with CD56 (clone 504, Novocastra, CA, USA), chromogranin (clone DAK-A3, Dako, Denmark), or synaptophysin (clone MRQ-40, Cell Marque, CA, USA) primary antibodies overnight at 4 °C, followed by incubation with horse-anti-mouse IgG (MP-7402, Life Technologies, Carlsbad, CA, USA) or horse anti-rabbit IgG (MP-7401, Vector Laboratories, CA, USA) secondary antibodies for 1 h at room temperature. Sections were then counterstained with Mayer’s hematoxylin (Sigma-Aldrich, St. Louis, MO, USA) and mounted with Permount mounting medium (SP15-100, Thermo Scientific, IL, USA). All incubations were conducted in dark, humid chambers. A positive signal was visualized using a microscope (Nikon2000U, Nikon, Tokyo, Japan).

### 4.3. Organoid Drug Tests

SPDTO #7 cells were used for the organoid cell viability assay. After dissociation of SCLC organoids into a single cell suspension using TrypLE Express, 200 cells in 10 μL of Matrigel were dispensed onto 96-well microplates and allowed to polymerize at 37 °C for 30 min. After 2 days, cisplatin and etoposide were added at concentrations of 0.01, 0.1, 1, 2, 5, or 10 μM for 8 days. Cell viability was assessed using CellTiter-Glo^®^ (Promega, WI, USA). To test the regrowth of organoids after cisplatin or etoposide treatment, the medium was replaced with fresh regular medium and organoids were monitored for a further 4 weeks. Brightfield images were captured using a TE-2000U microscope (Nikon, Tokyo, Japan). 

### 4.4. DNA Extraction and WES Analysis

DNA was extracted from frozen patient-derived tumor tissues and corresponding SCLC organoid samples using a DNeasy Blood and Tissue kit (Qiagen, Hilden, Germany) according to the manufacturer’s protocol. To generate standard exome capture libraries, an Agilent SureSelect Target Enrichment protocol for the Illumina paired-end sequencing library (Version C2, December 2018) was used with 1 µg input gDNA. In all cases, a SureSelect Human All Exon V6 probe set was used. The quantification of DNA and DNA quality was measured using PicoGreen and agarose gel electrophoresis. One microgram of genomic DNA from each cell line diluted in EB buffer was sheared to a target peak size of 150–200 bp using a Covaris LE220 focused-ultrasonicator (Covaris, Woburn, MA, USA) according to the manufacturer’s recommendations. An 8 microTUBE strip was loaded into the tube holder of the ultrasonicator to shear the DNA using the following settings: mode, frequency sweeping; duty cycle, 10%; intensity, 5; cycles per burst, 200; duration, 60 s × 6 cycles; temperature, 4–7 °C. The fragmented DNA was repaired, an “A” was ligated to the 3′ end, and Agilent adapters were ligated to the fragments. After confirming ligation, the adapter-ligated product was PCR-amplified. For exome capture, a 250 ng sample of DNA library was mixed with hybridization buffers, blocking mixes, RNase block and 5 µL SureSelect all exon capture library, according to the standard Agilent SureSelect Target Enrichment protocol. Hybridization to the capture baits was conducted at 65 °C using the heated thermal cycler lid option at 105 °C for 24 h in a PCR machine. The captured DNA was then washed and amplified. The final purified product was quantified via qPCR according to the qPCR Quantification Protocol Guide (KAPA Library Quantification kits for Illumina Sequencing platforms) and qualified using a TapeStation DNA screentape D1000 (Agilent, CA, USA). Sequencing was performed using the NovaSeq platform (Illumina, CA, USA). Genome analysis was performed as described previously [13,15]. The Genome Analysis Toolkit (GATK) v1.6.5. was used. Sequenced reads were aligned to the human reference genome (NCBI build 37) using the default options. A somatic variant calling for single nucleotide variants and short indels was performed with matched normal tissues using MuTect (v1.1.7) and SomaticIndelocator in GATK, respectively. Quality checks for fastq files were performed using FastQC (http://www.bioinformatics.babraham.ac.uk/projects/fastqc).

### 4.5. Statistical Analysis

All data are represented as mean ± standard deviation of at least three independent experiments. Statistical significance of differences was assessed. *p* values < 0.05 were considered statistically significant. Significance was denoted as n.s. not significant, * *p* < 0.05, ** *p* < 0.01, and *** *p* < 0.001.

## Figures and Tables

**Table 1:** The list of small cell lung cancer (SCLC) samples used for the formation of organoids.

CASE #	ORGANOID ID	SEX	AGE	LUNG CANCER TYPE	EP RESPONSE	THAWING TEST
**1**	SPDTO #1	Male	67	SCLC	◯	◯
**2**	SPDTO #2	Male	66	SCLC	◯	◯
**3**	SPDTO #3	Male	67	SCLC	◯	◯
**4**	SPDTO #4	Male	71	SCLC	◯	X
**5**	SPDTO #5	Male	53	SCLC	◯	◯
**6**	SPDTO #6	Male	65	SCLC	◯	◯
**7**	SPDTO #7	Male	69	SCLC	X	◯
**8**	SPDTO #8	Male	64	SCLC	◯	X
**9**	SPDTO #9	Male	73	SCLC	◯	◯
**10**	SPDTO #10	Male	35	SCLC	X	◯

## Data Availability

The data presented in this study are openly available at this paper.

## Supplementary Materials

The following are available online at https://www.mdpi.com/1422-0067/22/3/1349/s1, Figure S1: The characteristics of SCLC PDTO and the original tissues, Figure S2: The genetic characteristics of SPDTOs and the original tissues, and Figure S3: Validating a SPDTO#10 as a drug screening platform.
