# Supplementary material for: Establishment and Long-Term Expansion of Small Cell Lung Cancer Patient-Derived Tumor Organoids
Source: Int J Mol Sci. 2021 Jan 29;22(3):1349. doi: 10.3390/ijms22031349 (PMC7866263; doi:10.3390/ijms22031349)
Supplement: Supplementary file 1 [file ijms-22-01349-s001.pdf]

Supplementary Information

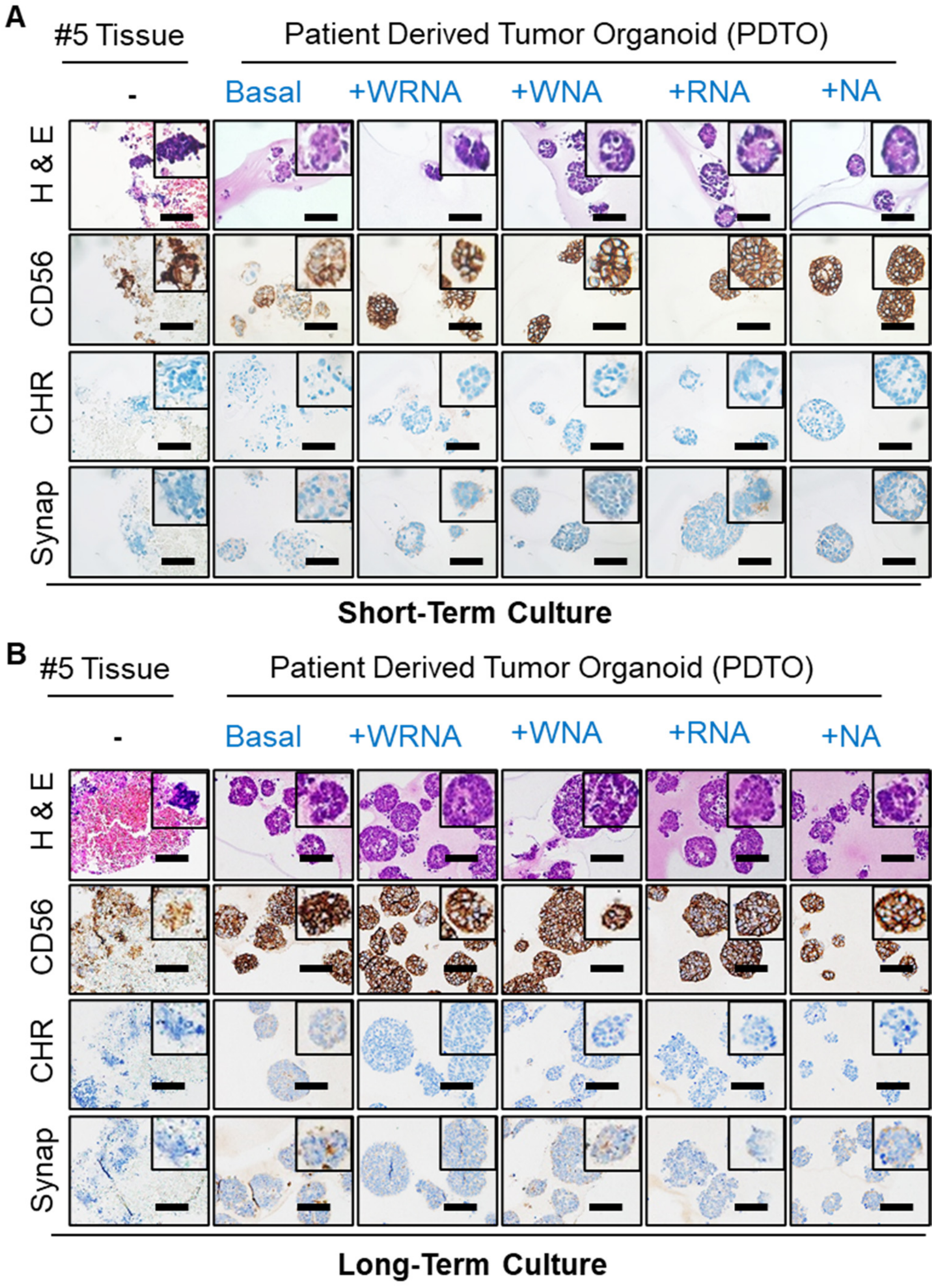

Supplementary Figure S1. The characteristics of SCLC PDTO and the original tissues

SPDTOs recapitulate the characteristics of the original tissues during short-term and long-term culture (A) H&E-stained and IHC-stained images of SPDTO #5 grown for short-term and their

original tissues. Scale bars represent 100 µm. (B) H&E stained and IHC-stained images of SPDTO #5 grown for long-term and their original tissues. Scale bars represent 100 µm

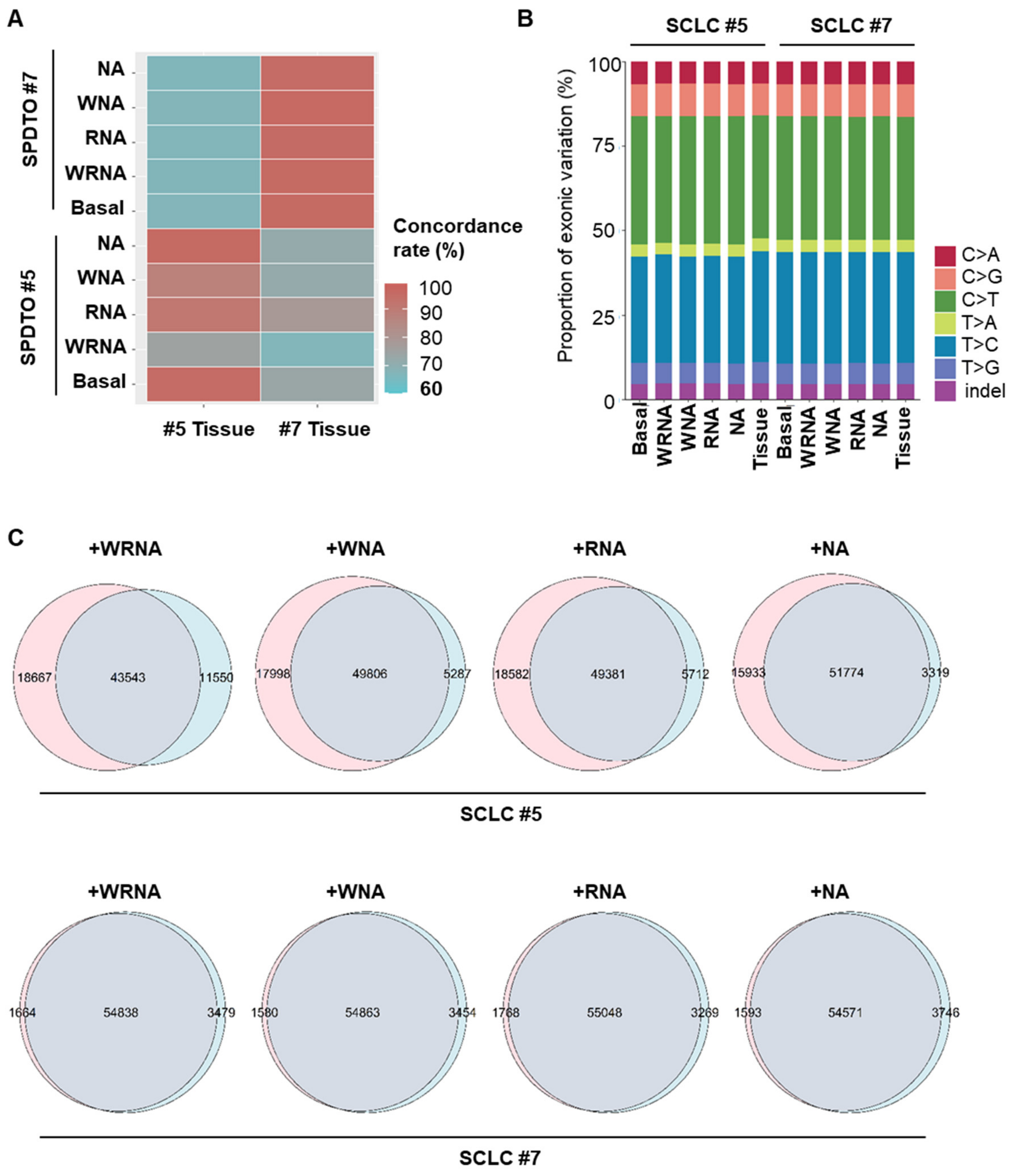

Supplementary Figure S2. The genetic characteristics of SPDTOs and the original tissues

SPDTOs preserve the genetic characteristics of the original tissues. (**A**) Correlation heat map

between SCLC tissues and paired SPDTOs (**B**) Proportions of variations across the samples, the six types of SNVs and the indels are represented. (**C**) Venn diagrams indicating the number of somatic mutations present in each SCLC tissues and their SPDTO.

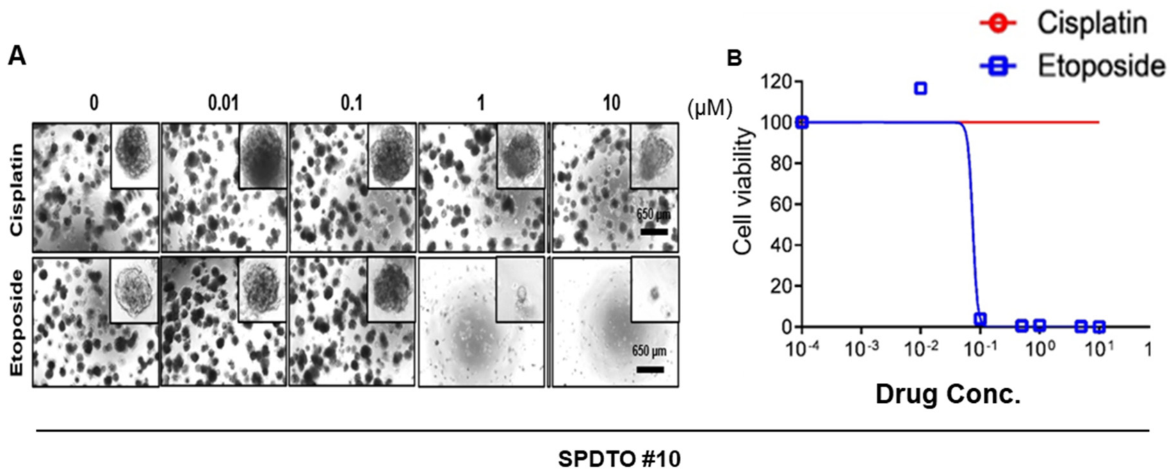

Supplementary Figure S3. Validating a SPDTO#10 as a drug screening platform. SPDTO #10 was treated with various concentrations of cisplatin or etoposide for 8 days. (A) Brightfield images of SPDTO #10 with 0, 0.01, 0.1, 1, 10 μM of cisplatin or etoposide treatments. (B) IC50 graph of cisplatin and etoposide in SPDTO #10
